# Novel strategies for HIV-1 epitope delivery using foamy viral hybrid proteins and vectors

**DOI:** 10.1186/1742-4690-9-S2-P321

**Published:** 2012-09-13

**Authors:** M Mühle, A Bleiholder, K Hoffmann, M Löchelt, J Denner

**Affiliations:** 1 Robert Koch Institute, Berlin, Germany; 2 German Cancer Research Center, Heidelberg, Germany

## Background

To prevent HIV-1 infection induction of broadly neutralising antibodies (bnAb) like 2F5 and 4E10 reacting with the membrane proximal external region (MPER) of the transmembrane envelope (TM) protein gp41 of HIV-1 may be essential. Since these antibodies appear late after infection, it is thought that they require a prolonged maturation time. Using apathogenic foamy virus (FV) as replicating gene delivery vector may allow antibody maturation through persistent antigen presentation. Based on studies showing an interaction of the MPER with the fusion peptide proximal region (FPPR) in gp41, strategies to graft these regions into backbone proteins of FV were examined and two, comprising the FV TM protein and the accessory Bet protein were tested here.

## Methods

The feline foamy virus (FFV) TM protein and Bet hybrid proteins comprising the MPER and FPPR of HIV-1 gp41 were produced and antibody responses were studied in immunized and FFV infected animals by ELISA, Western blot and microarray-based epitope mapping. Also, constructs for stable, high level expression of full length FFV Env were developed.

## Results

Mapping sera from animals immunized with the FFV TM protein and infected cats revealed immunogenic epitopes in the FV FPPR and MPER. These were exchanged against their HIV-1 homologues in optimized FV Env. Upon transfection, these constructs allowed production of chimeric virus-like-particles (VLPs) currently used for immunization studies. By immunizing with the Bet fusion proteins containing the gp41 MPER, FPPR or MPER-FPPR, strong responses were induced against gp41 and the 2F5 epitope ELDKWAS, demonstrating the feasibility of this approach. The hybrid sequences are now transferred to infectious FFV clones.

## Conclusion

To induce bnAb of the type 2F5 and 4E10, the developed vaccine systems provide two new high safety strategies for HIV epitope delivery by either VLPs or replicating vectors. This work was supported by the Volkswagen Foundation.

